# Attitudes toward Methadone among Out-of-Treatment Minority Injection Drug Users: Implications for Health Disparities

**DOI:** 10.3390/ijerph6020787

**Published:** 2009-02-23

**Authors:** Nickolas D. Zaller, Alexander R. Bazazi, Lavinia Velazquez, Josiah D. Rich

**Affiliations:** 1 The Miriam Hospital, 164 Summit Ave., Providence, RI 02906, USA; 2 Alpert Medical School, Brown University, Providence, RI 02912, USA; 3 Center for Prisoner Health and Human Rights, The Miriam Hospital, Providence, RI 02906, USA; 4 MAP Behavioral Health Services (MAP Outreach Program), 324 Elmwood Ave., Providence, RI 02907, USA

**Keywords:** Injection drug use, methadone, health disparities, HIV/AIDS

## Abstract

Injection drug use (IDU) continues to be a significant public health issue in the U.S. and internationally, and there is evidence to suggest that the burden of injection drug use and associated morbidity and mortality falls disproportionately on minority communities. IDU is responsible for a significant portion of new and existing HIV/AIDS cases in many parts of the world. In the U.S., the prevalence of HIV and hepatitis C virus is higher among populations of African-American and Latino injection drug users (IDUs) than among white IDUs. Methadone maintenance therapy (MMT) has been demonstrated to effectively reduce opiate use, HIV risk behaviors and transmission, general mortality and criminal behavior, but opiate-dependent minorities are less likely to access MMT than whites. A better understanding of the obstacles minority IDUs face accessing treatment is needed to engage racial and ethnic disparities in IDU as well as drug-related morbidity and mortality. In this study, we explore knowledge, attitudes and beliefs about methadone among 53 out-of-treatment Latino and African-American IDUs in Providence, RI. Our findings suggest that negative perceptions of methadone persist among racial and ethnic minority IDUs in Providence, including beliefs that methadone is detrimental to health and that people should attempt to discontinue methadone treatment. Additional potential obstacles to entering methadone therapy include cost and the difficulty of regularly attending a methadone clinic as well as the belief that an individual on MMT is not abstinent from drugs. Substance use researchers and treatment professionals should engage minority communities, particularly Latino communities, in order to better understand the treatment needs of a diverse population, develop culturally appropriate MMT programs, and raise awareness of the benefits of MMT.

## 1. Introduction

Injection drug use (IDU) continues to be a significant public health issue in the U.S. and internationally, and there is evidence to suggest that the burden of injection drug use and associated morbidity and mortality falls disproportionately on minority communities. In 96 of the largest U.S. metropolitan areas, the median prevalence of injection drug users in 2002 was estimated to be 96.1 per 10,000 people, with a great deal of variation in different metropolitan areas [1]. Though recent estimates suggest that black-white disparities in IDU have declined, IDU is still more prevalent among blacks, with white IDU prevalence in 2002 at approximately 90 per 10,000 adults and black IDU prevalence at 156 per 10,000 adults [2,3]. On average, rates of IDU are similar in Hispanic and non-Hispanic white populations, but disparities in IDU are present in some metropolitan areas [2], and the collection of data on Hispanics as a whole may obscure the significant variability between Hispanic subgroups [4,5]. U.S.-born Hispanics report higher rates of substance use than immigrants [6], and among Hispanics diagnosed with AIDS, those born in the U.S.A. or Puerto Rico were more likely to have contracted HIV through IDU than those born in Central or South America, Mexico, or Cuba [7]. Metropolitan areas with greater white-Latino differences in IDU tend to be located in the Northeast, an area where the majority of Latinos are of Puerto Rican origin [2]. In the Northeast, Latino-white disparities in IDU may be due to the relatively high rates of heroin use and high levels of residential segregation, which has been linked to increased IDU, found among Puerto Ricans living in mainland US [2,5,8].

Among injection drug users (IDUs), average HIV prevalence in the 96 largest U.S. metropolitan areas has been estimated to be 6.2% [9], far greater than the prevalence in the general population [10]. More than one in five people become infected with hepatitis C virus (HCV) within two years of beginning injection drug use, and more than one in three become infected within five years [11]. Hispanic IDUs are significantly more likely to be infected with HCV than non-Hispanic white or black IDUs [11,12]. Studies have shown also that HIV prevalence is higher among black and Latino IDUs than among white IDUs [13,14]. In addition to infectious disease risk, minorities are also at higher risk of dying from an overdose [15].

Racial and ethnic minorities bear a disproportionate share of drug-related morbidity and mortality, yet, as with other medical conditions in the U.S., they are less likely to receive the treatment services they need [16–18]. The drug treatment needs of different racial and ethnics groups vary significantly, as do the effects of treatment and ancillary social and health services [16]. African Americans and Latinos are less likely to receive services matched to their specific needs and are generally underserved by substance use treatment programs [16,19]. Data from a U.S. national survey indicates that a higher percentage of African Americans report a need for substance abuse treatment than whites [17]. Among individuals reporting a need to receive treatment for substance use or mental health disorders, whites are more likely than African Americans or Latinos to be receiving treatment, and African Americans and Latinos are more likely to report unmet need [17]. Additionally, Latinos report less satisfaction with all forms of substance use treatment and mental health care than Whites and African Americans [17].

Opiate replacement therapy is a widely used, effective treatment modality for opiate dependence. The most readily available form of opiate replacement therapy in the US is methadone maintenance therapy (MMT), which has been used to treat chronic heroin addiction for nearly 40 years [20,21]. Despite the demonstrated efficacy of MMT in reducing heroin usage [22,23], HIV risk behaviors[24] and transmission [25], overdose deaths [26], general mortality [27], and criminal behavior [28,29], MMT remains a treatment option that many opiate-dependent individuals do not utilize [20,30]. In a study of 28,000 IDUs accessing treatment in Massachusetts, Lundgren *et al.* found that Latinos and African Americans were more likely than whites to use detoxification-only treatment for drug addiction, a treatment modality that is generally cheaper and less effective [31,32] than MMT [33]. In this same study, African Americans were half as likely as whites to use this effective treatment [33].

Many treatment-seeking IDUs report not being able to enter treatment for reasons such as unavailability of treatment slots, prohibitively expensive costs or strict admission criteria [34,35]. But in addition to factors like cost and availability of treatment, the knowledge, attitudes and beliefs of drug users may influence whether and how often different groups access substance use treatment and what types of treatment they utilize. Misconceptions about methadone and ambivalence toward methadone treatment have been well documented [36,37]. In qualitative studies, opiate-dependent IDUs, particularly those not in treatment, generally report negative attitudes toward methadone [35,38]. Perceived negative side-effects of methadone, a dislike for the rigid requirements demanded by MMT and fear of withdrawal from methadone upon incarceration or involuntary discharge are also reported by opiate-dependent individuals as reasons for not entering MMT [35]. Common negative beliefs about methadone include the views that it is harmful to teeth and bones, that it is more damaging to health than heroin, and that withdrawing from methadone is nearly impossible [30]. Negative attitudes toward methadone can lead many patients to leave MMT programs prematurely [30], which can result in relapse to heroin use and a return to behaviors that put individuals at high risk for drug-related morbidity and mortality [22–25,27–29]. Among Latino IDUs in Philadelphia, Porter found that even though many individuals reported that MMT was beneficial in controlling their addiction, aspects of MMT programs, such as having to attend clinics daily and lack of adequate or culturally appropriate counseling services, were significant barriers to remaining in long-term treatment [39]. Even when minority IDUs enter treatment, many do not complete their treatment episodes. In her qualitative research with out-of-treatment Latino IDUs, Porter also found that 2/3 of out-of-treatment Latino IDUs had dropped out of at least one treatment program because they either did not like the program or were asked to leave due to a violation of program rules [39].

Given that methadone has been shown to be an effective treatment for opiate addiction and prevention strategy for the transmission of HIV and other infectious diseases, understanding why many IDUs do not currently participate in MMT merits further investigation. The diversity in populations of injection drug users makes understanding different racial and ethnic groups’ views about MMT crucial to designing interventions that are culturally appropriate, effective and function to improve access to opiate addiction treatment and to prevent IDU-related morbidity and mortality. In this article, we present data from surveys administered to out-of-treatment minority IDUs in Providence, RI.

## 2. Subjects and Methods

To collect data about perceptions of MMT among minority IDUs, between January 2007 and April 2008 we administered a brief survey to consenting out-of-treatment minority IDUs in Providence, RI. The survey was adapted from an instrument developed by Stancliff *et al.* to measure beliefs about methadone in an urban methadone clinic [30]. IDUs were recruited through participation at a free community HIV testing site and through street outreach by the city’s only needle exchange program, which is funded by the Department of Health and run by a local AIDS service organization. Individuals coming to MAP Outreach, a South Providence HIV testing and outreach site that provides evidence-based prevention interventions primarily to African American and Latino substance users, were offered the survey if they indicated IDU as a primary risk factor while being tested for HIV. AIDS Care Ocean State (ACOS) needle exchange outreach workers offered the survey to individuals they encountered while doing street-based syringe exchange with IDUs. The survey contained specific questions relating to constructs surrounding attitudes, beliefs and practices regarding methadone treatment. Interviews took approximately 15 minutes and all participants received a $10 food voucher for their participation.

### 2.1. Study Sample

We chose to target our recruitment efforts for this study on African American and Latino IDUs (the two most prevalent racial and ethnic minority populations in greater Providence) as insufficient data exists in Rhode Island about barriers and facilitators minority IDUs encounter in accessing MMT. For example, nearly 80% of all MMT patients in the state are Caucasian, while the total number of IDUs is much more heterogeneous. Our total sample consisted of 53 participants. While no statistical information was compiled regarding refusal rates, nearly everyone approached agreed to participate in the survey. The high acceptance rate was largely a function of the trusting relationships that clients had with the HIV testing agency and the street based needle exchange program. Homogenous sampling techniques were used to recruit IDUs to ensure that individuals were as similar as possible with respect to specific demographic characteristics.

### 2.2. Data Analysis

The prevalence of specific knowledge, attitudes and practices were determined. Participant demographics were tabulated and binomial probability analyses were performed to examine differences regarding specific survey responses. All survey responses were transformed into categorical variables and exact hypothesis testing was used to determine statistical significance between responses. All tests were two-sided and p-values < 0.05 were considered statistically significant. All statistical analyses were performed using STATA version 9. The Institutional Review Board of The Miriam Hospital approved the study protocol.

## 3. Results

### 3.1. Participant Characteristics

In the sample of 53 respondents, 11 (21%) were female and 41 (79%) were male, with an average age of 40 years. Fourteen respondents (26%) identified as non-Hispanic black and 39 (74%) identified as Hispanic. Among respondents identifying as Hispanic, the majority (79%) reported being of Puerto Rican origin. Thirty-two respondents (60%) reported past experience with methadone therapy. Participant characteristics are detailed in Table 1.

### 3.2. Knowledge, Attitudes and Beliefs

Respondents generally expressed negative attitudes toward methadone. Most respondents (73%, p<0.01) believed or were uncertain that methadone was bad for a person’s health. Seventy-seven percent of respondents (p<0.01) believed that people should attempt to discontinue methadone treatment. Most respondents believed that methadone programs are too expensive (70%, p=0.01) and also that coming to a methadone clinic for treatment makes life difficult (72%, p<0.01).

Sixty-six percent of respondents (p=0.03) believed that being on methadone means that a person is not abstinent from drugs, and 70% (p=0.01) perceived that people in recovery look down upon people on methadone therapy. Despite generally negative attitudes toward methadone therapy, 62% of respondents (p=0.01) believed that it would helpful for opiate users to receive methadone at doctors’ offices. Participant responses are detailed in Table 2.

## 4. Discussion

Potential barriers to treatment identified by this study include beliefs about the negative effects of methadone on health, the legitimacy of MMT as a drug treatment modality, and the cost of MMT. These findings are consistent with recent research in Baltimore among a predominantly African-American population of IDUs that has identified similar barriers to treatment entry as well as generally negative perceptions of methadone, particularly among out-of-treatment individuals [35,38]. A potentially important barrier to either initiating or continuing MMT is the belief that methadone is harder to withdraw from than heroin, which was emphasized in writing by several study participants. One participant wrote that methadone “makes you sicker when you stop” and another commented, “After getting out of methadone, it was worse than the drug habit. I had to use heroin again and then go to detox”.

Though not statistically significant, some differences were found in the responses of African Americans and Latinos, with more Latinos believing that methadone brings shame on a person’s family and also that people at methadone clinics do not understand different cultures or minority issues. These differences highlight the need to develop a better understanding of the obstacles encountered by Latinos, which may include language and cultural barriers to accessing treatment. An important component of providing appropriate substance use and other health-related treatment services to Latino communities is understanding cultural context and recognizing barriers to enrollment and retention as well as delivering or linking to comprehensive services [40].

It is interesting to note that in the neighboring state of Massachusetts, Reynoso-Vallejo *et al.* found that Puerto Rican men were more likely than other male Latino IDUs to utilize methadone maintenance [41]. Yet negative attitudes persist among Puerto Rican IDUs included in our study. There is significant variability even within racial and ethnic sub-groups, and to develop a full understanding of the treatment needs of specific populations of IDUs, the effects of specific factors like geographic location, place of birth and degree of acculturation must be examined [5,6].

### Limitations

Limitations of this study include the small sample size and potential for bias in the sampling procedure, as the potential for self selection was present among individuals accessing HIV testing or needle exchange services. Participants entered the study through either a syringe exchange or HIV testing program and may not be representative of the general IDU population in Providence. Compared to local demographics, Puerto Ricans are overrepresented in the study population. However, anecdotal information from outreach workers suggests that Puerto Ricans are over-represented in the Providence IDU population, which is consistent with national findings about substance use among Latino sub-populations[5,7]. It is possible that social desirability bias also affected survey responses. Due to the above limitations, the results of this study may not be generalizable to specific groups in Providence or other regions of the US and should be interpreted with caution. Additionally, even though this study focuses exclusively on MMT, efforts should be made to understand barriers to enrolling minority IDUs in buprenorphine maintenance treatment, another effective form of opiate replacement therapy.

## 5. Conclusion

Our study highlights the need for targeted education about MMT for out-of-treatment minority drug users, including education in Spanish to effectively reach the underserved population of Latino IDUs. More research is needed to understand specific cultural attitudes toward addiction and addiction treatment in many Latino and African-American communities. Further research should address the questions of how misconceptions about methadone can be dispelled and how treatment providers can become more responsive to minority, particularly Latino, populations. It addition, MMT clinics should be more engaged in understanding and addressing the specific needs of minority IDUs that might be uncomfortable or unable to communicate with clinic staff.

The gravity of the negative social and health consequences often accompanying opiate addiction, including HIV and HCV infection, demands a stronger response from researchers and drug treatment providers to more effectively enroll minority IDUs in opiate addiction treatment. Developing a better understanding of minority attitudes toward MMT and using this understanding to reduce racial and ethnic disparities in MMT utilization has the potential to mitigate inequalities in opiate addiction and drug-related morbidity and mortality in racial and ethnic minority populations.

## Figures and Tables

**Table 1.** Participant characteristics.

Total, n (%)	53 (100%)
**Age**	
20 – 29 y	6 (12%)
30 – 39 y	19 (37%)
40 – 49 y	21 (41%)
50 – 59 y	5 (10%)
**Gender**	
Female	11 (21%)
Male	41 (79%)
**Race/Ethnicity**	
Black, non-Hispanic	14 (26%)
Hispanic (any race)	39 (74%)
**Place of Birth**	
USA	14 (29%)
Puerto Rico	31 (63%)
Other or Multiple	4 (8%)
Drug(s) of Choice*	
Heroin	34 (64%)
Cocaine/Crack	5 (9%)
Heroin and Cocaine/Crack	14 (26%)
**Utilize Syringe Exchange Programs**	
Yes	27 (51%)
No	26 (49%)
Source of Needles**	
Individuals	13 (27%)
Syringe Exchange	20 (41%)
Pharmacy	20 (41%)
Other	4 (8%)
**Past Methadone Treatment**	
Yes	32 (60%)
No	21 (40%)

* All participants indicated heroin use, but survey responses reflect their primary drug of choice.

** Percents do not add up to 100 since participants could indicate more than one response.

**Table 2.** Attitudes and Beliefs.

**Methadone is bad for a person’s health.**			
Agree or Don’t Know	38 (73%)	
Disagree	14 (27%)	<0.01
**People should try to get off methadone.**			
Agree	41 (77%)	
Disagree or Don’t Know	12 (23%)	<0.01
**Methadone treatment prevents HIV and/or HCV.**			
Agree	20 (38%)	
Disagree or Don’t Know	33 (62%)	0.09
**Being on methadone means a person isn’t abstinent from drugs.**			
Agree	35 (66%)	
Disagree or Don’t Know	18 (34%)	0.03
**People in recovery look down on those on methadone.**			
Agree	37 (70%)	
Disagree or Don’t Know	16 (30%)	0.01
**Coming to methadone clinic makes life difficult.**			
Agree	38 (72%)	
Disagree or Don’t Know	15 (28%)	<0.01
**Methadone programs are too expensive.**			
Agree	37 (70%)	
Disagree or Don’t Know	16 (30%)	0.01
**People would be helped if methadone were offered in doctors’ offices.**			
Agree	33 (62%)	
Disagree or Don’t Know	20 (38%)	0.01
**Being on methadone brings shame on a person’s family.**			
Agree	18 (37%)	
Disagree or Don’t Know	31 (63%)	0.09
**There should be family counseling at methadone clinics.**			
Agree	42 (86%)	
Disagree or Don’t Know	7 (14%)	<0.01
**Clinics don’t understand different cultures or minority issues.**			
Agree	28 (58%)	
Disagree or Don’t Know	20 (42%)	0.32
